# Retraction: Transplantation of *HGF* gene-engineered skeletal myoblasts improve infarction recovery in a rat myocardial ischemia model

**DOI:** 10.1371/journal.pone.0294365

**Published:** 2023-11-08

**Authors:** The PLOS ONE Editors

Following the publication and Correction of this article [1, 2], concerns were raised regarding results presented in Figs 3, 4, 7, and 8. Specifically,

- In Fig 3a, the SM-Only Day3 panel appears to partially overlap with the SM-Only Day14 panel.
- In Fig 4e, the SM-Only panel appears to partially overlap with the SM-GFP panel when rotated 180°.
- In Fig 7a, the SM-Only panel and the GFP panels appear more similar than would be expected from independent results.
- In Fig 8a, there appear to be repetitive elements within each panel, as well as repetitive elements between panels.

The corresponding author provided a response and underlying data for Figs 3, 4, 7, and 8, but these did not resolve the concerns raised with the article. In light of the concerns affecting multiple figure panels that question the integrity of these data, the *PLOS ONE* Editors retract this article.

SLR, CYZ, ZHS, LHC, XFH, HJD, and BL either did not respond directly or could not be reached. XLW and XJL responded but expressed neither agreement nor disagreement with the editorial decision.
